# Integrative Transcriptomic and Proteomic Profiling Identifies PMEL as a Critical Regulator of Melanogenesis in Rex Rabbits

**DOI:** 10.3390/ani15213135

**Published:** 2025-10-29

**Authors:** Shuaishuai Hu, Jingwen Zhang, Pei Zhang, Mingyan Shi, Ying Zhang

**Affiliations:** Life Science College, Luoyang Normal University, Luoyang 471934, China; 13721616449@163.com (J.Z.); zhangpei8877@126.com (P.Z.); mingyanshi@lynu.edu.cn (M.S.)

**Keywords:** rex rabbits, melanin, transcriptome, proteome, PMEL

## Abstract

**Simple Summary:**

Coat color is a significant production trait in fur-bearing animals, having a substantial impact on their economic value. The diverse coat colors in rex rabbits provide a valuable resource for investigating the genetic mechanisms underlying coat color formation. In this study, we performed integrated transcriptomic and proteomic profiling of skin tissues from black and white rex rabbits, identifying PMEL as an important regulator of melanogenesis. Our findings demonstrate that PMEL plays a crucial role in melanin synthesis, melanocyte proliferation, and the expression of melanin-related genes, thus shedding light on the genetic basis of coat color variation in rex rabbits. Our results suggest that PMEL contributes to melanogenesis in rex rabbits. We believe that our study will be of great interest to the readers of *Animals*, given its implications for understanding the genetic control of coat color diversity in fur-bearing animals.

**Abstract:**

Coat color is a crucial production trait for fur-bearing animals and significantly influences their economic value. The remarkable diversity of coat colors in rex rabbits not only provides a wide range of market options but also serves as an essential resource for investigating the genetic mechanisms underlying coat color formation. In this study, we conducted integrated transcriptomic and proteomic profiling of skin tissues from black and white Rex Rabbits, revealing the presence of 52 co-expressed genes/proteins. Proteomic analysis revealed a significant upregulation of PMEL (*p* = 0.030, FC = 2.194), while transcriptomic data indicated an even more pronounced upregulation (*p* = 0.028, FC = 35.279). Therefore, PMEL (Premelanosome Protein) may serve as a pivotal regulator of melanogenesis in Rex Rabbits. Our findings indicate that PMEL overexpression in melanocytes increases melanin content, promotes melanocyte proliferation, and enhances the expression of melanin-related genes (*MITF*, *TYR*, *TYRP1*, and *GPNMB*) while inhibiting melanocyte apoptosis. Conversely, PMEL knockdown significantly reduces melanin content, melanocyte proliferation, and the expression of melanin-related genes while promoting melanocyte apoptosis. These findings suggest that PMEL contributes to melanogenesis in Rex Rabbits.

## 1. Introduction

Coat color, one of the most prominent phenotypes in mammals, represents a phenotypic trait of profound economic and scientific importance [1]. In the fur industry, variations in coat color directly influence market value, with specific coat colors commanding premium prices. Beyond their economic significance, coat color serves as a critical model for studying pigmentation and genetic regulation, providing insights into melanocyte development, melanin synthesis, and evolutionary adaptations.

Numerous studies have confirmed that coat color is influenced by multiple mechanisms, including gene expression regulation, protein function execution, and intercellular signal interaction. Melanin serves as the decisive factor in determining coat color, with its synthesis governed by a conserved regulatory network. The process of melanin synthesis is highly coordinated process. MITF, as the central regulatory factor, activates the expression of key genes such as TYR and TYRP1, which determine both the type and quantity of melanin produced, subsequently influencing the formation of mammalian coat color [2,3,4,5]. Furthermore, the Wnt/β-catenin, SCF/c-KIT, and MAPK signaling pathways, along with certain non-coding RNAs, regulate the expression of key genes that affect melanin production [6,7,8,9]. The aforementioned research indicates that key genes can affect melanin synthesis through various regulatory patterns. Pigment deposition is intricately linked to the functional state of melanocytes, the type of melanin produced, and the spatial distribution of these pigments within the skin [10]. This process is co-regulated by genetic factors and external environmental influences. For instance, the loss-of-function variant of the MC1R gene is closely associated with the phenotypes of red hair and fair skin [11]. Individuals carrying such variants not only demonstrate a diminished capacity to synthesize melanin in response to ultraviolet radiation but also exhibit heightened sensitivity to skin photodamage. In contrast, individuals with darker skin tones benefit from the superior light scattering and absorption properties of eumelanin, which confer enhanced intrinsic photoprotection and significantly lower the risk of developing malignant skin tumors [12]. Pathogenic mutations in genes associated with melanosomes, such as TYR, TYRP1, and DCT, can result in oculocutaneous albinism, characterized by hereditary defects in the melanin biosynthesis pathway [13]. Furthermore, dysregulation of signaling between melanocytes and keratinocytes within the epidermal melanin unit may also precipitate acquired pigmentary disorders, including melasma and post-inflammatory hyperpigmentation [14]. These pathological conditions can have profound negative effects on patients’ mental health and social functioning.

Transcriptomics and proteomics, as core technologies in systems biology, provide essential tools for elucidating the molecular mechanisms underlying coat color formation through comprehensive analyses of gene expression and protein dynamics. Several studies utilizing transcriptome analysis have identified that the differences in skin color among human epidermal melanocytes are primarily driven by the regulatory expression of the *SLC45A2* gene, rather than the traditionally acknowledged enzyme tyrosinase. Furthermore, research has unveiled novel regulatory networks, including orphan receptors and chlorine channels, which present new therapeutic targets for pigmentary disorders [15]. Systematic investigations have elucidated the critical roles of *SLC45A2* and *GPNMB* genes in melanin deposition in chicken feathers through transcriptome sequencing and functional validation [16]. Additionally, transcriptome analysis has demonstrated that melanin deposition in the pectoral muscles of Xuefeng black-bone chickens is synergistically regulated by core genes associated with the tyrosine metabolic pathway (*TYR*, *TYRP1*, and *DCT*) and melanosome structure genes (*GPNMB* and *MLPH*). This finding provides molecular targets and a theoretical basis for enhancing poultry meat quality [17]. Moreover, transcriptome sequencing has indicated that the coat color of cashmere goats is regulated by a balance between *TYR*/*TYRP1*/*DCT*/*PMEL* (which promotes melanogenesis) and *ASIP*/*AHCY* (which suppresses melanogenesis), identifying key target genes for the molecular breeding of cashmere goats and contributing to the enhancement of their economic traits [18]. Furthermore, previous studies have systematically unveiled the molecular mechanisms underlying the differences in black and white coat color in minks (Neovison vison) through a combined transcriptomic and proteomic analysis. Key genes such as *KITLG*, *LEF1*, *DCT*, *TYRP1*, *PMEL*, *Myo5a*, *Rab27a*, and *SLC7A11* have been identified as regulators of coat color formation, providing molecular targets for mink coat color breeding [19]. Additionally, key differential proteins, including APOA1 and FGA, have been identified in sheep through skin proteomics sequencing, which offers targets for genetic breeding related to coat color [20]. Furthermore, integrated transcriptome and proteome analyses revealed that the expression peaks of genes such as MED23 and FZD10 were synchronized with the molting cycle at 8 weeks of age, confirming their involvement in the temporal regulation of feather pigmentation. This research not only provides molecular targets for poultry breeding but also offers a new perspective for studying pigment diseases [21]. Collectively, these studies demonstrate that integrated transcriptomics and proteomics can comprehensively analyze the molecular mechanisms of mammalian coat color formation, transitioning from gene expression to protein function. They reveal the roles of core genes such as *MITF*, *PMEL*, and *TYR*, while also providing key targets for fur color genetic improvement and the treatment of human pigment disorders.

Rex Rabbit (Oryctolagus cuniculus) is a representative fur-type rabbit, renowned for its superior fur quality, high density, and diverse array of coat colors. Garments made from Rex Rabbit fur provide excellent warmth retention and vibrant colors, which are highly favored by consumers. As living standards improve and the fur industry develops, the demand for Rex Rabbit fur in various colors has been increasing both domestically and internationally. Therefore, the development of new coat color varieties in Rex Rabbits is of substantial practical importance. Understanding the genetic basis of coat color variation is essential for selective breeding and offers insights into human pigmentary disorders such as albinism and vitiligo. To elucidate the mechanisms underlying the formation of different coat colors in Rex Rabbits, we collected skin tissues from both black and white Rex Rabbits for transcriptomic and proteomic sequencing. Through a combined multi-omics analysis, we identified the key regulatory factor PMEL. Subsequently, we verified the impact of PMEL on melanogenesis in Rex Rabbits. This research provides a theoretical basis for the genetic improvement of coat colors in Rex Rabbits and contributes to the study of pigment-related diseases.

## 2. Materials and Methods

### 2.1. Animals and Samples Collection

Six-month-old black and white Rex Rabbits of comparable weight and age were randomly selected as either male or female from a rex rabbit breeding facility in Luoyang, Henan Province. All rabbits were raised under identical environmental conditions. For each coat color, three rabbits were selected, euthanized, and anesthetized. The dorsal fur was shaved clean using a depilator agent, and dorsal skin samples measuring 1 cm^2^ were collected from the same area. The samples were immediately placed in liquid nitrogen and subsequently transferred to a −80 °C freezer for storage to be used for total RNA and protein extraction.

### 2.2. Transcriptomic Sequencing

Total RNA was extracted from the skin using RNAsimple Total RNA kit (Tiangen Biotech Co., Ltd., Beijing, China) according to the manufacturer’s instructions. The quality of RNA was assessed using 5300 Bioanalyser (Agilent, Santa Clara, CA, USA) and quantification was performed with the ND-2000 (Thermo, Waltham, MA, USA). RNA purification, reverse transcription, library construction and sequencing were performed at Shanghai Majorbio Bio-pharm Biotechnology Co., Ltd. (Shanghai, China) according to the manufacturer’s instructions. The RNA-seq transcriptome library from the skin of Rex Rabbits was prepared using Illumina^®^ Stranded mRNA Prep, Ligation (San Diego, CA, USA) using 1 μg of total RNA. Briefly, messenger RNA was extracted using the polyA selection method utilizing oligo (dT) beads and subsequently fragmented with fragmentation buffer. Double-stranded cDNA was synthesized using random hexamer primers. The resulting cDNA underwent end-repair, phosphorylation, and adapter attachment according to the library construction protocol. Size selection of the libraries for cDNA target fragments ranging from 300 to 400 bp was carried out using magnetic beads, followed by PCR amplification for 10 to 15 cycles. After quantification with Qubit 4.0, sequencing libraries were prepared on the DNBSEQ-T7 platform (PE150) utilizing the DNBSEQ-T7RS Reagent Kit (FCL PE150) version 3.0.

The raw paired-end reads underwent trimming and quality control using fastp [22] with the default settings. Clean reads were then aligned to the reference genome in orientation mode using HISAT2 (v2.1.0) [23] software. The assembled mapped reads for each sample were processed using StringTie (v2.1.2) [24] in a reference-based manner.

To determine the differential expression genes (DEGs) between two distinct samples, the expression levels of individual transcripts were calculated using the transcripts per million reads (TPM) approach. Gene abundances were quantified using RSEM [25]. Differential expression analysis was conducted with DESeq2 [26]. The criterion of fold change > 1.5 or <0.67 and *p*-value < 0.05 were classified as significantly differentially expressed genes. Furthermore, functional enrichment analyses, including Gene Ontology (GO) and Kyoto Encyclopedia of Genes and Genomes (KEGG), were performed to ascertain which DEGs were significantly enriched in specific GO terms and metabolic pathways, using a Bonferroni-corrected *p*-value < 0.05 relative to the whole-transcriptome background. GO enrichment analysis of DEGs was performed across the three major categories: biological process (BP), cellular component, and molecular function, as classified in the GO framework. GO functional enrichment and KEGG pathway assessments were executed using Goatools (v0.6.5) and Python (v3.11)’s scipy library, respectively.

### 2.3. Proteomics Sequencing

We removed the skin tissue samples from the frozen state and placed them on ice. The samples were suspended in a protein lysis buffer containing 8 M urea and 1% SDS, along with a commercially available protease inhibitor cocktail (used at a 1:100 dilution) to prevent protease activity. The mixture was treated in a high-flux tissue grinder for three cycles, each lasting 180 s. Subsequently, non-contact cryogenic sonication was conducted for 30 min. The precipitate was then dissolved in a protein lysate solution that included 8M urea, 1% SDS, and a cocktail of protease inhibitors. Following 2 min of sonication on ice, the mixture was centrifuged at 12,000× *g* for 20 min at 4 °C, and the protein concentration in 1 μL of the supernatant was quantified using the ProteoAnalyzer (Agilent, Santa Clara, CA, USA) with bovine serum albumin (BSA) as the standard curve.

100 μg protein was re-suspended in Triethylammonium bicarbonate buffer (TEAB) at a final concentration of 100 mM. The mixture was reduced with Tris (2-carboxyethyl) phosphine (TCEP) at a final concentration of 10 mM at 37 °C for 60 min, followed by alkylation with iodoacetamide (IAM) at a final concentration of 40 mM at room temperature for 40 min in the dark. After centrifugation at 10,000× *g* at 4 °C for 20 min, the pellet was collected and re-suspended with 100 μL TEAB at a final concentration of 100 mM. Trypsin was added at a 1:50 trypsin-to-protein mass ratio and incubated at 37 °C overnight.

After trypsin digestion, the peptides were dried using a vacuum pump. Subsequently, the enzymatically drained peptides were re-solubilized in 0.1% trifluoroacetic acid (TFA) and desalted using HLB. The resulting peptides were then dried with a vacuum concentrator. Finally, the peptides were quantified using the NANO DROP ONE (Thermo, MA, USA) based on UV absorption values.

Peptide quantification results were analyzed using a Vanquish Neo system connected to an Orbitrap Astral mass spectrometer (Thermo, USA) at Majorbio Bio-Pharm Technology Co., Ltd. (Shanghai, China). In summary, an Orbitrap Astral mass spectrometer operating in DIA mode was employed to acquire data-independent acquisition (DIA) data. Briefly, the uPAC High-Throughput column (75 μm × 5.5 cm, Thermo, USA) was used with solvent A (water with 2% ACN and 0.1% formic acid) and solvent B (water with 80% ACN and 0.1% formic acid). The chromatography run time was set to 8 min. Data-independent acquisition (DIA) data were acquired using an Orbitrap Astral mass spectrometer operated in DIA mode. The mass spectrometry scanning range was set from 100 to 1700 *m*/*z* for the full scan, with a resolution of 60,000, followed by data-dependent acquisition (DDA) of the top 20 most intense ions for MS/MS fragmentation. To analyze the DIA raw data, spectronaut software (Version 19) was implemented. The specifications included: a peptide length range of 7–52; trypsin/P as the enzyme cutting site; a maximum of 2 missed cleavage sites; with carbamidomethylation of cysteines as a fixed modification along with oxidation of methionines and protein N-terminal acetylation as variable modifications. The thresholds were set to a protein FDR of ≤0.01, peptide FDR of ≤0.01, peptide confidence of ≥99%, and XIC width of ≤75 ppm. Protein quantification was performed using the MaxLFQ approach.

For the bioinformatic analysis of the proteomic data, the Majorbio Cloud platform (https://cloud.majorbio.com) (accessed on 24 July 2025) was utilized [21]. The R package “*t*-test” was employed to calculate *p*-values and Fold change (FC) between the two groups for the proteins. The criteria for identifying differentially expressed proteins (DEPs) included fold change thresholds of > 1.2 or < 0.83, in addition to *p*-value < 0.05. Functional annotation for all identified proteins was conducted using Gene Ontology (GO) (http://geneontology.org/) (accessed on 24 July 2025) and KEGG pathways (http://www.genome.jp/kegg/) (accessed on 24 July 2025). The DEPs were subsequently analyzed for GO and KEGG enrichment. For protein–protein interaction analysis, String v11.5 was used.

### 2.4. Cell Culture and Transfection

The isolation and culture of melanocytes were performed following the methodology described by Chen et al. [27]. Rabbit melanocytes were maintained in Melanocyte Medium-2 (MelM-2, ScienCell, Carlsbad, CA, USA) at 37 °C with 5% CO_2_. To ensure the stability of phenotypic and functional characteristics, only passages 3 to 5 of melanocytes were utilized in this study. The cultures of melanocytes were routinely tested for mycoplasma contamination and confirmed to be negative prior to use in experiments. The testing was performed using the PCR method in accordance with standard laboratory protocols. Cell transfections were carried out when the cells reached approximately 80% confluency utilizing the Lipomaster 3000 Transfection Reagent (Vazyme, Nanjing, China). For transfection, either a PMEL overexpression plasmid (2 μg/well for a 6-well plate) or siRNA targeting PMEL (50 nM final concentration) was used. Cells were harvested 48 h post-transfection for subsequent RNA and protein extraction, which were employed for the functional identification of PMEL.

### 2.5. Vector Construction

Total RNA was isolated from melanocytes using the RNAsimple Total RNA kit (Tiangen Biotech Co., Ltd., Beijing, China) according to the manufacturer’s instructions. First-strand cDNA synthesis was conducted using HiScriptII QRT SuperMix (Vazyme, Nanjing, China). Cloning primers for PMEL (NCBI Reference Sequence: NM_001297728.1) were designed using CE Design software (V3) (Vazyme, Nanjing, China), and subsequently inserted into pcDNA3.1 vectors employing restriction enzymes Nhe I and EcoR I. The coding sequence (CDS) of PMEL was cloned using the ClonExpress II One Step cloning kit (Vazyme, Nanjing, China) (see Supplementary Table S1).

### 2.6. Melanin Content Detection

The method used to assess melanin content was conducted in accordance with previously published literature [28]. Melanocytes transfected in a 6-well plate were washed three times with phosphate-buffered saline (PBS) and then detached using trypsin for cell collection. Subsequently, the cells were treated with 1 M sodium hydroxide (NaOH) and incubated in a water bath at 80 °C for 1 h to solubilize the melanin. The resulting samples were transferred into 96-well plates, and a microplate reader was employed to measure the absorbance at a wavelength of 475 nm. The relative melanin content was determined by normalizing the absorbance values of the treatment group against those of the control group. To account for potential variations in cell number, the melanin content was normalized to the total protein content. An aliquot of the cell suspension was taken prior to NaOH addition for protein quantification using a bicinchoninic acid (BCA) protein assay kit (Thermo Scientific, USA). The final melanin content was expressed as A475 per μg of total protein.

### 2.7. Quantitative Real-Time PCR Analysis

The expression levels of fifteen differentially expressed genes (DEGs) and genes related to melanin synthesis, were evaluated using the ChamQ Universal SYBR qPCR Master Mix (Vazyme, Nanjing, China) through quantitative reverse transcription PCR (qRT-PCR). Detailed information regarding the specific primer sequences employed in the analysis of these DEGs is provided in Supplementary Table S1. Each sample was analyzed in three independent trials to ensure the robustness and reliability of the results. The data were normalized using GAPDH as the reference standard for accurate comparison and interpretation of gene expression levels. The relative mRNA expression levels were calculated using the 2^−ΔΔCt^ method [29].

### 2.8. Western Blotting (WB)

Tissues and cells were lysed to extract total proteins using RIPA buffer, which was supplemented with phenylmethylsulfonyl fluoride (PMSF) and a phosphatase inhibitor. The concentrations of these proteins were measured using the BCA Protein Assay Kit (Thermo Scientific, USA). Following this, protein samples underwent electrophoresis on a 12% sodium dodecyl sulfate-polyacrylamide gel (SDS-PAGE) (Bio-Rad, Shanghai, China). The proteins were subsequently transferred to polyvinylidene fluoride (PVDF) membranes (Merck Millipore, Burlington, MA, USA). To reduce non-specific binding, the membranes were incubated with 5% non-fat milk for an hour. An overnight incubation at 4 °C with primary antibodies was then conducted, as detailed in Supplementary Table S2. After this step, the PVDF membranes were treated with the appropriate secondary antibodies for one hour. Visualization of protein bands was accomplished using the Bio-Rad ChemiDoc imaging system (Hercules, CA, USA).

### 2.9. Cell Proliferation and Apoptosis Assay

24 h post-transfection, melanocytes were harvested and distributed into 96-well plates, with each well containing 100 μL of cell suspension. The cells were subsequently cultured for 0, 24, 48, and 72 h. To evaluate cell proliferation, each well was incubated with 10 μL of Cell Counting Kit-8 (CCK-8) solution (Vazyme, Nanjing, China). After a 4 h incubation, the absorbance of the samples was measured at 450 nm using a microplate reader. For the assessment of cell apoptosis, samples were processed using the Annexin V-FITC apoptosis detection kit (Vazyme, Nanjing, China) according to the manufacturer’s instructions. The rate of cell apoptosis was determined through flow cytometry analysis (FACSAria SORP, Becton Dickinson, Franklin Lakes, NJ, USA). The apoptosis rate was calculated using the following equation: total number of cells is composed of number of cells in the right upper quadrant and number of cells in the right lower quadrant.

### 2.10. Statistical Analysis

Bioinformatic analysis of proteomic data was conducted using the Majorbio Cloud platform (https://cloud.majorbio.com) (accessed on 24 July 2025). *p*-values and Fold change (FC) for the proteins between the two groups were calculated using R package (version 2.15) “*t-*test”. Additional statistical analyses were conducted using SPSS version 25 (SPSS Inc., Chicago, IL, USA) and GraphPad Prism 9.0. Statistical significance between two groups was assessed using an unpaired Student’s *t*-test, whereas comparisons among more than two groups were conducted using one-way analysis of variance (ANOVA) followed by Tukey’s post hoc test. The experimental group and the control group had three replicates each. Results are presented as mean ± standard deviation (SD). The *p*-value < 0.01 indicates extremely significant differences, while the *p*-value < 0.05 indicates significant differences.

## 3. Results

### 3.1. Transcriptomic Analysis of the Black and White Groups

**Figure 1:**
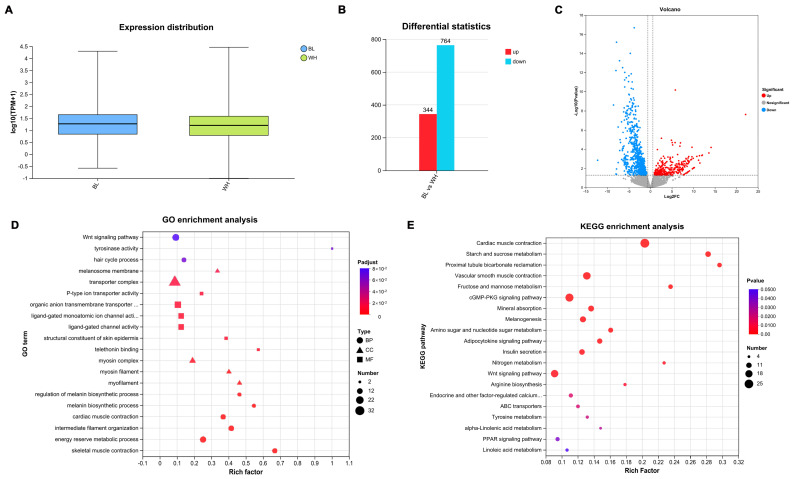
Transcriptomic analysis of DEGs in skin from the black vs. white rex rabbits. Reference gene source: Oryctolagus_cuniculus; Reference genome version: OryCun2.0. The experimental group and the control group had three replicates each. (**A**) Expression distribution of transcriptome data across all six samples (*n* = 3 per group). The box plots illustrate the overall gene expression consistency within and between groups. (**B**) The number of upregulated (344) and downregulated (764) DEGs identified between black and white rex rabbit skin tissues in transcriptome data. (**C**) Volcano plot visualizing the distribution of DEGs. (**D**) GO analysis of DEGs across biological process, cellular component, and molecular function categories. The top 20 significantly enriched terms are shown. (**E**) KEGG analysis of DEGs. The top 20 significantly enriched pathways are displayed.

To comprehensively analyze the key genes responsible for color differences between black and white rex rabbits, a transcriptomic analysis was performed. The results indicated that over 40.03 Mb of clean reads were obtained from the six cDNA libraries (Supplementary Table S3). The Q30 for each sample exceeded 95.88%, with approximately 83.76% of the clean reads uniquely mapped to the rabbit genome (Oryctolagus_cuniculus 2.0). Overall, the gene expression trends were consistent across the six skin samples from both black and white rex rabbits (Figure 1A). These data suggest high-quality sequencing, thus ensuring the reliability of results for further investigation.

To identify pivotal genes involved in the formation of coat color in rex rabbits, we applied the criterion of fold change > 1.5 or < 0.67 and *p*-value < 0.05. This approach resulted in the identification of 1108 DEGs, comprising 344 upregulated and 764 downregulated DEGs between the black and white groups (Figure 1B). Additionally, the volcano plot of DEGs clearly illustrates the distribution of upregulation and downregulation among the identified 1108 DEGs (Figure 1C). Detailed information regarding these DEGs is provided in Supplementary Data S1.

GO enrichment analysis revealed that DEGs are predominantly enriched in biological processes associated with the Wnt signaling pathway, tyrosinase activity, and melanin synthesis; cellular components including the melanosome membrane, transport complex, and myosin complex; and molecular functions related to hair formation, and epidermal structural components of the skin (Figure 1D). Furthermore, KEGG enrichment analysis indicated significant enrichment in the Wnt signaling pathway, GMP-PKG signaling pathway, melanogenesis and tyrosine metabolism (Figure 1E). Moreover, the majority of GO and KEGG distributions were associated with immunity, growth development, and metabolism. The results suggest that the identified DEGs play extensive regulatory roles in the coat color of rex rabbits.

### 3.2. Validation of DEGs by qPCR

**Figure 2:**
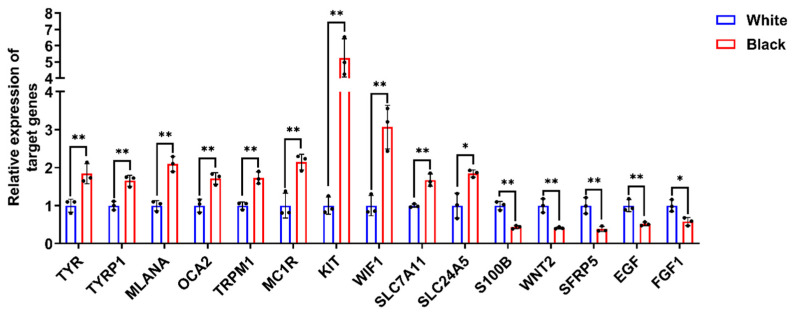
The verification of DEGs expression by RT-qPCR. RT-qPCR was conducted utilizing gene-specific primer pairs designed for target gene amplification. Relative expression was calculated using 2^−ΔΔCt^ method with GAPDH as internal control. Data were expressed as mean ± standard deviation (S.D.). Each experiment was conducted three times in repetition. ** *p* < 0.01, * *p* < 0.05.

To further validate the reliability of the transcriptome data, we performed RT-qPCR analysis on 15 genes associated with melanogenesis: tyrosinase (*TYR*), tyrosinase related protein 1 (*TYRP1*), melan-A (*MLANA*), melanosomal transmembrane protein (*OCA2*), transient receptor potential cation channel subfamily M member 1 (*TRPM1*), melanocortin 1 receptor (*MC1R*), proto-oncogene, receptor tyrosine kinase (*KIT*), WNT inhibitory factor 1 (*WIF1*), solute carrier family 7 member 11 (*SLC7A11*), solute carrier family 24 member 5 (*SLC24A5*), S100 calcium binding protein B (*S100B*), Wnt family member 2 (*WNT2*), secreted frizzled related protein 5 (*SFRP5*), epidermal growth factor (*EGF*), and fibroblast growth factor 1 (*FGF1*) from both black and white groups to validate the expression levels obtained from the transcriptome data. The results of RT-qPCR were consistent with those from RNA-Seq (Figure 2), demonstrating the accuracy and authenticity of the skin RNA-Seq results.

### 3.3. Proteomic Analysis of the Black and White Groups

**Figure 3:**
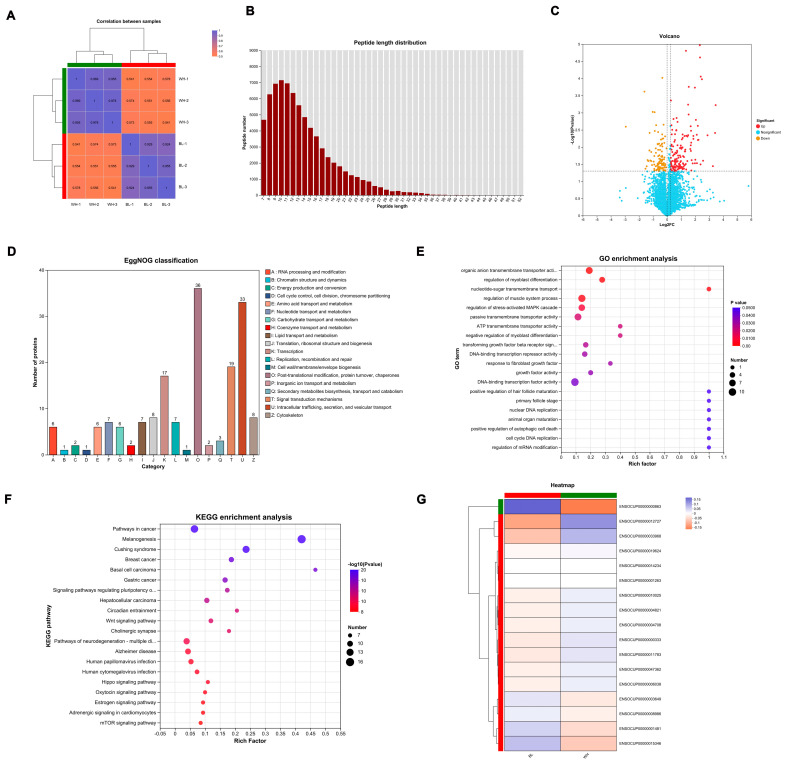
Proteomic profiles of skin from black and white rex rabbits. (**A**) Correlation analysis of the samples from black and white groups. (**B**) The length distribution of most peptides. (**C**) Volcano plot of DEPs. (**D**) EggNOG categories of DEPs. (**E**) GO enrichment of DEPs across biological process, cellular component, and molecular function categories. The top 20 significantly enriched terms are shown. (**F**) KEGG pathway analysis of DEPs. The top 20 significantly enriched pathways are displayed. (**G**) Cluster analysis of DEPs.

Proteomic sequencing was conducted to further investigate the mechanisms underlying the coat color differences between black and white rex rabbits. As shown in Figure 3A, the results of the correlation analysis were clustered within their respective groups, indicating a more pronounced discrimination. A total of 79,442 peptides were identified, with the lengths of the detected peptides mainly being distributed between 7 and 25 amino acids (Figure 3B). This distribution characteristic aligns with the principles of tryptic digestion. The peptide length distribution detected by mass spectrometry meets the quality assessment criteria for mass spectrometry data. Differentially expressed proteins (DEPs) were screened using the fold change thresholds of >1.2 or <0.83 and *p*-value < 0.05. A total of 313 DEPs were identified between the black and white group, comprising 108 downregulated and 205 upregulated proteins, with a volcano plot clearly illustrating the distribution of DEPs (Figure 3C). According to EggNOG classification (Figure 3D), the number of DEPs was relatively high in the functional classifications of O (Post-translational modification, protein turnover, chaperones), U (Intracellular trafficking, secretion and vesicle transport), and T (Signal transduction mechanisms). To further elucidate the biological functions of the DEPs, Gene Ontology (GO) and Kyoto Encyclopedia of Genes and Genomes (KEGG) analyses were performed. GO analysis revealed that the DEPs are primarily involved in processes such as ATP transmembrane transporter activity, DNA-binding transcription factor activity, animal organ maturation, nuclear DNA replication, growth factor activity, and hair follicle maturation (Figure 3E). KEGG analysis of the DEPs indicated enrichment in the Wnt signaling pathway and melanogenesis (Figure 3F), both of which are known to be associated with melanin synthesis. Additionally, cluster analysis revealed that certain DEPs, such as ENSOCUP00000000863 (PMEL), ENSOCUP00000012727 (WNT3), and ENSOCUP00000033968 (DVL3), exhibited significant differences between the black and white groups. All DEPs are listed in Supplementary Data S2.

### 3.4. Combined Analysis of Transcriptomics and Proteomics

**Figure 4:**
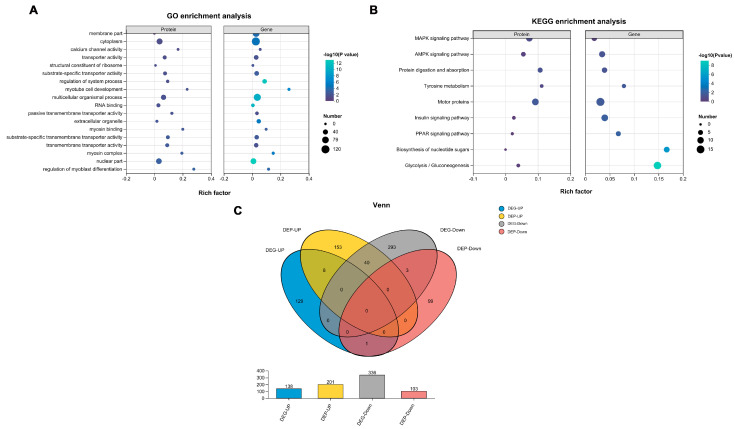
Combined analysis of transcriptomics and proteomics of skin from black and white rex rabbits. (**A**) GO enrichment analysis of DEGs and DEPs. (**B**) KEGG pathway analysis of DEGs and DEPs. (**C**) Venn analysis of DEGs and DEPs.

To further explore the mechanisms underlying the melanogenesis in rex rabbits, a combined analysis of transcriptomics and proteomics was conducted. GO analysis revealed a significant enrichment of various terms, primarily associated with the cytoplasm, transporter activity, and regulation of systemic processes (Figure 4A). Additionally, KEGG analysis of the DEPs indicated an enrichment in pathways related to protein digestion and absorption, tyrosine metabolism, and motor proteins (Figure 4B). Subsequently, Venn analysis was performed to identify factors shared between transcriptomics and proteomics, revealing the presence of 52 co-expressed genes/proteins (Figure 4C). All data are available in Supplementary Data S3.

### 3.5. PMEL, a Crucial Regulator, Promotes Melanin Synthesis in Melanocytes

**Figure 5:**
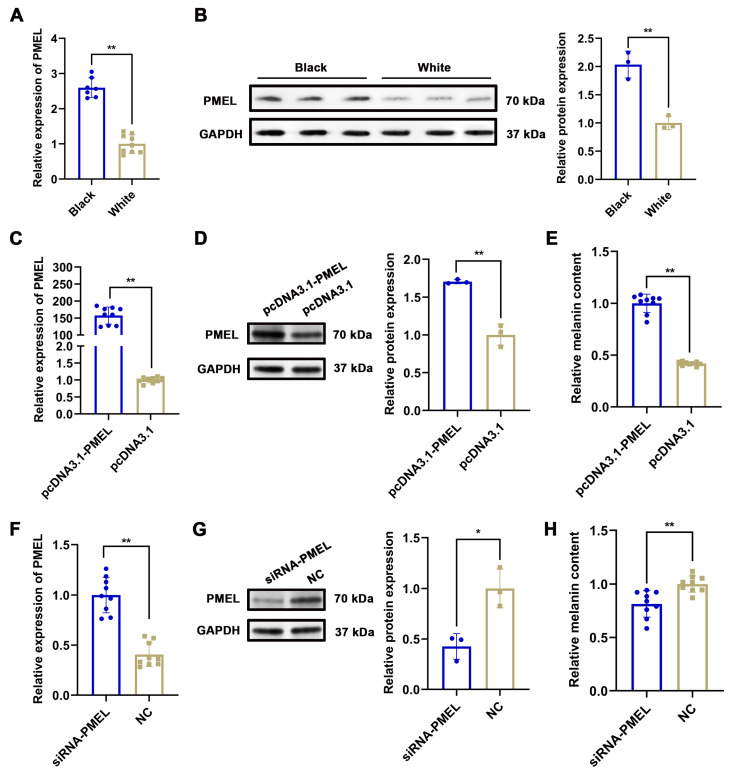
The effect of PMEL on melanin synthesis in melanocytes. (**A**,**B**) The mRNA and protein expression of PMEL in skin tissues of black and white rex rabbits were detected by RT-qPCR and WB in skin of black and white rex rabbits, respectively. GAPDH was used as an internal control. (**C**) and (**D**) The mRNA and protein expression of PMEL were detected by RT-qPCR and WB after PMEL was overexpressed in melanocytes, respectively. (**E**) Melanin content was detected by NaOH after PMEL overexpression in melanocytes. (**F**,**G**) The mRNA and protein expression of PMEL were detected by RT-qPCR and WB after PMEL knockdown in melanocytes, respectively. (**H**) Melanin content was detected by NaOH after PMEL knockdown in melanocytes. ** *p* < 0.01, * *p* < 0.05.

To identify the key factors regulating melanogenesis in Rex Rabbits, we analyzed studies related to 52 proteins (including PMEL, SMARCD3, CLCA2, S100G, KLHL31, STAC3, etc.) through a comprehensive literature search. Our analysis revealed that PMEL is significantly associated with melanogenesis [30]. Furthermore, proteomic analysis demonstrated a notable upregulation of PMEL (*p* = 0.030, FC = 2.194), while transcriptomic data indicated an even more pronounced upregulation (*p* = 0.028, FC = 35.279). Consequently, we hypothesized that PMEL acts as a crucial regulator in melanogenesis. Our results demonstrated that the mRNA and protein expression levels of PMEL in the skin of black Rex Rabbits were significantly higher than those in white Rex Rabbits (*p* <0.01), corroborating the transcriptome sequencing results (Figure 5A,B). To elucidate the effect of PMEL on melanin synthesis, we measured melanin content following PMEL overexpression and knockdown in melanocytes. The results indicated a significant increase in both mRNA and protein expression of PMEL (*p* < 0.01), along with a marked increase in melanin content (*p* < 0.01) (Figure 5C–E). Conversely, PMEL knockdown resulted in a significant decrease in melanin content (*p* < 0.01) (Figure 5F–H). These findings suggest that PMEL plays a positive regulatory role in melanogenesis.

### 3.6. PMEL Enhances the Proliferation of Melanocytes While Inhibiting Apoptosis

**Figure 6:**
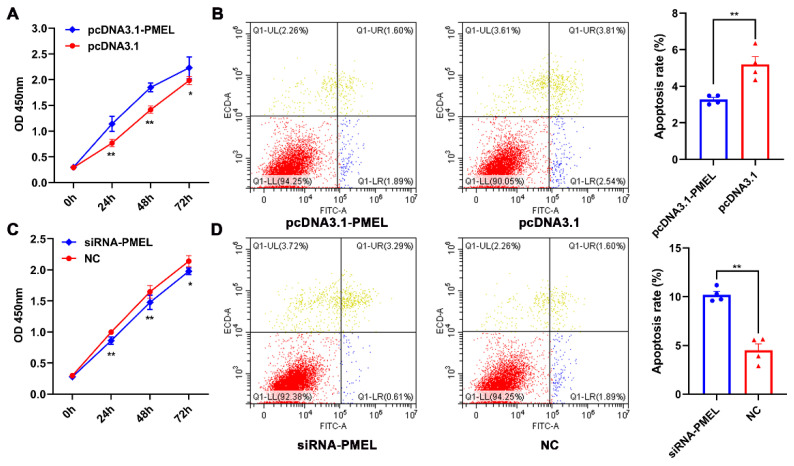
The regulatory effect of PMEL on the proliferation and apoptosis of melanocytes. (**A**,**B**) The proliferation and apoptosis of melanocytes were estimated in response to PMEL overexpression in melanocytes. (**C**,**D**) The proliferation and apoptosis of melanocytes was determined after PMEL knockdown in melanocytes. ** *p* < 0.01, * *p* < 0.05.

To verify the regulatory effect of PMEL on melanocytes, we assessed the proliferation and apoptosis levels using the CCK-8 and FITC/PI methods during PMEL overexpression and knockdown. Our results indicated that PMEL overexpression significantly increased the proliferation of melanocytes while inhibiting apoptosis (*p* < 0.01) (Figure 6A,B). Conversely, PMEL knockdown resulted in inhibited proliferation and increased apoptosis (*p* < 0.01) (Figure 6C,D). These results highlight the importance of PMEL in regulating both the proliferation and apoptosis of melanocytes.

### 3.7. PMEL Influences the Expression of Key Genes in the Melanogenesis Pathway

**Figure 7:**
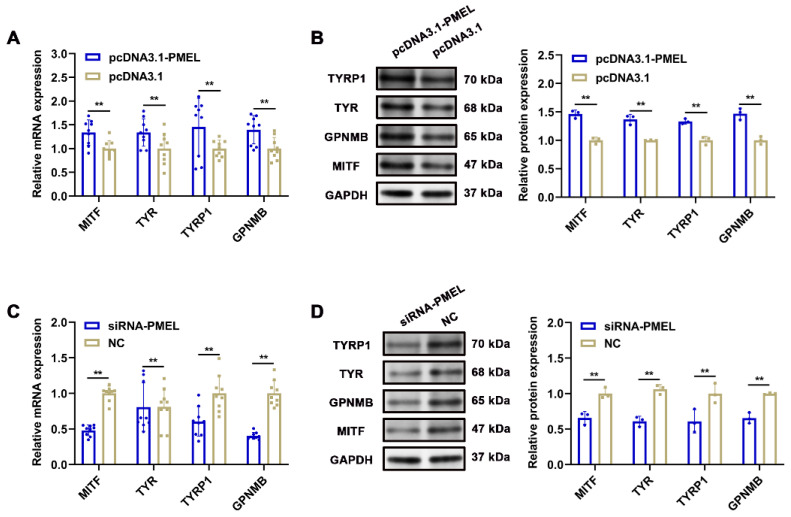
The regulatory effect of PMEL on melanin-related genes. (**A**,**B**) The mRNA and protein expression levels of melanin-related genes were detected after PMEL overexpression in melanocytes. (**C**,**D**) The mRNA and protein expression levels of melanin-related genes were detected after PMEL knockdown in melanocytes. ** *p* < 0.01.

To further investigate the regulatory impact of PMEL on the melanogenesis pathway, we measured the mRNA and protein levels of melanin-related genes, including *MITF*, *TYR*, *TYRP1*, and *GPNMB*, in response to PMEL overexpression and knockdown in melanocytes. Our findings revealed that PMEL overexpression significantly increased the mRNA and protein levels of these genes (*p* < 0.01) (Figure 7A,B). Conversely, following PMEL knockdown, the mRNA and protein levels of these genes were significantly reduced (*p* < 0.01) (Figure 7C,D). These results indicate that PMEL regulates melanin synthesis by modulating the expression of melanin-related genes.

## 4. Discussion

The coat color of mammals possesses significant economic value. Elucidating the molecular mechanisms underlying the formation of coat color in Rex Rabbits is profoundly significant for both fundamental biological research and practical applications, including genetic improvement and breeding strategies in domestic animals, as well as biomedical research on pigmentary disorders. This study aims to unravel the molecular mechanisms driving coat color differences between black and white rex rabbits through integrated transcriptomic and proteomic approaches. Both transcriptomic and proteomic analyses revealed significant enrichment in melanogenesis, tyrosine metabolism, and Wnt signaling. The findings indicate that melanogenesis and tyrosine metabolism are crucial in the process of coat color formation in fur-bearing animals. These pathways are well-documented in melanocyte biology, where they coordinate melanin production, melanosome biogenesis, and melanocyte differentiation [31]. It should be noted that tyrosine metabolism, a crucial pathway, serves as the metabolic backbone of melanin synthesis. Tyrosine is converted to L-DOPA by TYR, which represents a rate-limiting step in eumelanin production [32]. Additionally, in the Wnt signaling pathway, some genes indirectly regulate melanin production by influencing the expression of MITF, such as Wnt5a, WNT1 and WNT3a [33,34]. By integrating transcriptomic and proteomic datasets, we identified 52 co-expressed genes/proteins, including PMEL, SMARCD3 (a chromatin remodeler), and S100G (a calcium-binding protein), etc. These candidates likely form a regulatory network that coordinates melanocyte development, melanin synthesis, and melanosome transport. Future studies utilizing CRISPR-Cas9 or overexpression techniques could validate their roles in coat color determination.

It is worth noting that our findings not only illuminate key genes, signaling pathways, and cellular processes involved in melanin synthesis but also emphasize that PMEL could play a significant role in melanogenesis. PMEL (Premelanosome Protein), a scaffolding glycoprotein, is critical for melanosome maturation and melanin deposition. It forms amyloid fibrils that template melanin polymerization, and its dysfunction results in hypopigmentation across vertebrates [35,36]. While its expression correlates with melanosome maturation [37], its functional role in coat color regulation remains ambiguous in Rex Rabbits. Our results indicate that mRNA and protein levels of PMEL are significantly higher in black rabbit skin compared to white rabbits, suggesting that PMEL may be involved in melanin synthesis. Functional validation through overexpression and knockdown in melanocytes demonstrated that PMEL directly enhances melanin content. Furthermore, PMEL promotes melanocyte proliferation and inhibits apoptosis, while upregulating critical genes for melanosome biogenesis and melanin polymerization, including *MITF*, *TYR*, *TYRP1*, and *GPNMB*. The PMEL signaling peptide domain is highly conserved among vertebrates, and mutations in cattle, horses, dogs and chickens all result in color fading. PMEL not only regulates pigment synthesis but also directly influences the transformation of color patterns in crawling organisms from spots to stripes by controlling the spatial distribution of pigment progenitor cells during the embryogenesis [38]. Studies have identified that the PMEL gene, as a core driver of coat color variation, harbors missense mutations (such as Hypotrichosis_PMel17) that alter amino acid sequences, directly impacting melanin deposition [39]. The interaction between the PMEL signaling peptide regional mutation (P. Leu18del) and the *MC1R* gene co-regulates the six coat colors of highland cattle, providing new insights into the genetic mechanisms underlying coat color in domestic cattle [40]. Meanwhile, the PMEL P. Leu18del deletion mutation serves as the genetic basis for the dilution of coat color in Japanese brown cattle. Its incomplete dominance and cross-breed conservation render it an effective DNA marker for controlling coat color breeding [41]. Furthermore, biallelic truncating variants in the PMEL gene have been identified in humans, leading to a novel form of oculocutaneous albinism (OCA), characterized by a unique age-dependent pigment recovery phenomenon [42]. Additionally, PMEL upregulation in black rabbits may create a positive feedback loop; increased PMEL enhances melanosome maturation, which in turn amplifies signals (e.g., via Wnt pathways) that stabilize MITF and other melanogenic transcription factors [43]. As is well known, MITF serves as master transcription factor regulating melanocyte survival, proliferation, and the expression of pigmentation genes (e.g., *TYR*, *DCT*, *PMEL*, and *GPNMB*) [44,45]. Our results indicate that PMEL regulates melanogenesis by modulating MITF expression.

The aforementioned research elucidates the robust correlation between PMEL levels and coat color intensity that we observed, suggesting that PMEL indeed plays a significant regulatory role in pigment deposition. Our findings are highly consistent with this notion.

However, this study does have limitations. Our small sample size (n = 3 per group) restricts generalizability; expanding to larger, genetically diverse populations would enhance the robustness of our conclusions. Additionally, our focus on skin tissue neglects systemic signals (e.g., hormones) that may influence coat color. In vivo validation, such as generating PMEL transgenic/knockout rabbits, would further solidify causal links. Lastly, interactions between PMEL and pathways, such as MC1R, which modulates eumelanin/pheomelanin ratios, remain unexplored and warrant further investigation.

## 5. Conclusions

In summary, our integrated omics approach has identified PMEL as a key regulator of melanogenesis in rex rabbits, influencing melanocyte proliferation, apoptosis, and the expression of melanin-related genes. Our results provide preliminary molecular evidence that could inform future breeding research in domestic rabbits and other mammals. Moreover, our findings reveal hidden regulatory networks that contribute to phenotypic variation, thereby bridging fundamental research and applied innovation through the emphasis of specific economic traits.

## Data Availability

The data presented in this study are available in Supplementary Materials.

## Supplementary Materials

The following supporting information can be downloaded at: https://www.mdpi.com/article/10.3390/ani15213135/s1, Supplementary Table S1: The information for primers used in this study; Supplementary Table S2: The information of primary antibody; Supplementary Table S3: Clean reads analyses in white and black groups; Supplementary Data S1: Differentially expressed statistics in the transcriptome; Supplementary Data S2: Differentially expressed statistics in the proteome; Supplementary Data S3: Differentially expressed statistics between transcriptome and proteome.
