# Supplementary material for: Integrative Transcriptomic and Proteomic Profiling Identifies PMEL as a Critical Regulator of Melanogenesis in Rex Rabbits
Source: Animals (Basel). 2025 Oct 29;15(21):3135. doi: 10.3390/ani15213135 (PMC12609467; doi:10.3390/ani15213135)
Supplement: Supplementary file 1 [file animals-15-03135-s001.zip › animals-3881969-supplementary Tables.pdf]

Supplementary Table S1. The information for primers used in this study.

Name	Primer sequence (5' → 3')
pcDNA3.1(+)-PMEL	F: gggagacccaagctggctagcATGGACCTGGTGCTGAGAGGG<br>R: tgctggatatctgcagaattcTCAGACCTGCTGTCCACCGA
siRNA-NC	F: UUCUCCGAACGUGUCACGUtt<br>R: ACGUGACACGUUCGGAGAAtt
SiRNA-PMEL-982	F: CCUACUUCAGAAGUCCUAATT<br>R: UUAGGACUUCUGAAGUAGGTT
PMEL	F: CTGTCTGATATACAGGCGCAGA<br>R: CCAGAGAGATCAAGGCGTCA
TYR	F: GGGATAGCAGATGCCACTCAA<br>R: CAACAAACGCATGGTGAAGGA
TYRP1	F: TCCGTCTTCTCTCAATGGCG<br>R: GCCCATTCTCAGTGCTGTTAC
MLANA	F: CCAATGCTCCACCTGCCTAT<br>R: CAGGTGAGTTGCTGGCTCTTA
OCA2	F: GAATATGTCGGAGAGCAGAC<br>R: GAAGCACAGGAATCATTGTAG
TRPM1	F: CTCCTCATGTGGACAGCTCC<br>R: CAAGCTTTCTGGCCCTGC
MC1R	F: TACACCCCATGGCTCAGTTG<br>R: CCACTCACCATTGTCCACCA
KIT	F: CACACAGAAGATGGGGCTCG<br>R: ATCTCGTTGAGTGCGAGCTG
WIF1	F: AGAGCAGTGTGAAATCAGCAA<br>R: GGCTCCATACCGCTTTGAAC
SLC7A11	F: TCACCATTGGCTACGTGCT<br>R: GCCACAAAGATCGGAACTGCT
SLC24A5	F: CCTCCGTGAAGCTGAACACT<br>R: CTGGGTGCCGTTTCCTGT
S100B	F: TCAAAGAGCAGGAGGTTGTGG<br>R: TAACCATAGCGACGAAAGCCA
WNT2	F: AGTCTGACCTGATGCGGAC<br>R: CTGTAGCTCTCATGTACCACCAT
SFRP5	F: CGCAGTGTGAGATGGAGCA<br>R: TGCGCATTTTGACCACAAAGTC
EGF	F: AGGATTGACCCAGAAGGAACA<br>R: TATATTGCACACTCTCTCTTGCC
FGF1	F: CTTTTATACGGCTCGCAAACG<br>R: CCGTTTTTCTTCAGCCCCAC
GAPDH	F: CACCAGGGCTGCTTTTAACTCT<br>R: CTTCCCGTTCTCAGCCTTGACC

The complete insert sequence of PMEL (NCBI Reference Sequence:

NM_001297728.1):

at ggacctggtg ctgagagggt gccttctcca
61 cttggctggg atgagtgtcc tgctggctgc agggggcgca gaaggatcca cagaccggga
121 ctggcttggt gcctcaagga agctcagagc taaagcgtgg aacaggcagc tgtacccaga
181 gtggacagaa gcccagaggt ctgactgttg gagaggtggc caggtgtccc tgaaggtcag
241 caatgatggg cctacactga ttggtgcaaa cgcgtcctt c tccatcgccc tgcactttcc
301 tgaaagccaa aaggtactgc cagatgggca aattgtctgg gccaacaaca ccactctcaa
361 cgggagccag gtgtggggag gacagccagt gtatccccag gagcctgatg atggctgtgt
421 cttccctgat ggtggcccct gcccacttgg ccctttgtct cagaggagga gcttcgttta
481 cgtctggaag acctggggcc aatactggca agttctaggg ggcccagtgt ccaggctgag
541 cattgggaca gcccaggcga tgctgggcac acacaccatg gaggggactg tctaccaccg
601 ccgggggccc cagagctatg tgccccttgc ccactccagc tcagccttca cagtgactga
661 ccaggtgccc ttctctgtga gtgtgtccca gctgcaggcc ttggatggag ggaacaagcg
721 cttcctgaga aaccagcctc tgacttttgc catccagctc catgacccca gtggctatct
781 ggatggggct gacctctcct acacctggga cttcggagac gggactggaa ccctgatctc
841 tcgggctctt gtggtcactc acacttacct ggagcctggc cccgtcactg cccaggtggt
901 gctgcaggcg gccatccctc tcatctcctg tggctcctcc ccagttccag gcaccacaga
961 tgggtacttg ccaacgtcag gtgcccctgg caccacagct gggcaagggc ctacttcaga
1021 agtcctaagt accacacctg gtcaggtgtc aactacagag ccctctggaa ccacagcagc
1081 cataagtact gcacctgtgc aggtgccaac tacacagggc acagatacca cacccaagca
1141 gctgtcaacc tcccagggca cagtcaccac atctgcagac atgtcaacta ctgaggctat
1201 gggtacgact cctgcagagg tgttcactgg agagctctct ggagccacac ttgcgcaggt
1261 cacagctaca gagctggtag acacgccagc tggagaatta cccacccctg agcctgaggc
1321 tccagatgcc agcccattca tgcctacaga agtcattaca ggctccctgg gcagcccccct
1381 gctggatggc acagcctcgt taacgctggt gaagagacaa gtgcctctgg actgtgtgct
1441 ctatcgatac ggttcctact ccctcaccct cgacattgtc cagggcattg agaatgctga
1501 gatcctgcag gctgtgccat ccagtgagag ggatgcattt gagctgactg tctcctgcca
1561 aggcgggctg ccccaggaag cttgtatgga catctcatca ccagggtgcc agcccccctgc
1621 ccagcggctg tgccagcctg tgccgcccag cccagcctgc cagctggttc tgcaccaggt
1681 actgaatggc ggctcaggga cctactgcct caatgtgtcc ttggctgacg ccaacagcct
1741 ggcagtggtc agcacccagc ttgtcatgcc tggtcaagag gcaggcctgg gacaggcccc
1801 tctgtttgtc gggatcttgc tggggttgat ggccgtgggg cttgcctgtc tgatatacag
1861 gcgcagacta atgaagcagg gctcggctct ccccactccc cagctgccac cccgcggcac
1921 ccactggctg cgtctgccgc gggtcttccg tgcttgccct gctggtgaga acagccccct
1981 cctcggtgga cagcaggtct ga

Supplementary Table S2. The information of primary antibody.

**Primary antibody**	**Code**	**Producer**	**Dilution ratio**	**Band size（kDa）**
GAPDH	60004-1-Ig	Proteintech	1:500	37
MITF	sc-52938	SANTA CRUZ	1:1000	47
GPNMB	66926-1-Ig	Proteintech	1:10000	65
TYR	31291-1-AP	Proteintech	1:1000	68
TYRP1	sc-58437	SANTA CRUZ	1:500	70
PMEL	sc-393094	SANTA CRUZ	1:500	70

Supplementary Table S3. Clean reads analyses in white and black groups.

Sample	Raw reads/Mb	Clean reads/Mb	Q30(%)	Total mapped rate/%	Uniquely mapped rate/%
BL_1	43.73	43.48	96.41	93.12%	88.92%
BL_2	40.31	40.03	96.37	91.68%	88.47%
BL_3	43.14	42.82	96.09	89.99%	85.4%
WH_1	45.25	44.96	96.26	91.76%	87.95%
WH_2	45.34	45.04	96.39	92.52%	88.85%
WH_3	44.10	43.74	95.88	89.08%	83.76%
